# Application of the AHA-Proposed Cancer-Related Ischemic Stroke Classification: Reclassification and Prognostic Impact

**DOI:** 10.1161/STROKEAHA.126.055707

**Published:** 2026-06-03

**Authors:** Yasufumi Gon, Tomohiro Kawano, Hiroshi Yamagami, Tsutomu Sasaki, Manabu Sakaguchi

**Affiliations:** Department of Neurology, Graduate School of Medicine, The University of Osaka, Japan (Y.G., T.K., T.S., M.S.).; Department of Medical Innovation, Academic Clinical Research Center, The University of Osaka Hospital, Japan (Y.G.).; Division of Stroke Prevention and Treatment, Institute of Medicine, University of Tsukuba, Japan (H.Y.).; StemRIM Institute of Regeneration-Inducing Medicine, Graduate School of Medicine, The University of Osaka, Japan (T.S.).; Department of Neurology, Osaka General Medical Center, Japan (M.S.).

**Keywords:** atherosclerosis, ischemic stroke, neoplasms, prognosis, survival rate, thrombosis

## Abstract

**BACKGROUND:**

Ischemic stroke in patients with active cancer is heterogeneous and frequently classified as cryptogenic under the TOAST classification (Trial of ORG 10172 in Acute Stroke Treatment). The American Heart Association recently proposed an etiological classification for cancer-related ischemic stroke (CRIS). This study aimed to evaluate its clinical and prognostic implications.

**METHODS:**

We analyzed data from the prospective SCAN study (Ischemic Stroke in Patients With Cancer and Neoplasia), which enrolled patients with acute ischemic stroke and active cancer in Japan. Among the registered patients, those with available D-dimer data were included in the analysis. Stroke subtypes initially classified according to the TOAST criteria were reclassified using the CRIS framework. Kaplan-Meier survival curves were constructed, and differences were assessed using the log-rank test.

**RESULTS:**

Of 135 enrolled patients, 132 (median age, 75; 37.9% female) were included. Under the TOAST classification, 9 patients had small vessel occlusion, 20 large artery atherosclerosis, 28 cardioembolism, 10 other determined cause, and 65 cryptogenic strokes. After reclassification, 2 patients originally categorized as other determined etiology due to disseminated intravascular coagulation, and 46 patients previously classified as cryptogenic stroke were reclassified as CRIS. Patients classified as CRIS demonstrated significantly worse 1-year survival than those classified as conventional etiologies or cryptogenic stroke (global log-rank, *P*<0.001). The 3-month survival rate was 37.5% in the CRIS group and 89.2% in the reclassified cryptogenic stroke group.

**CONCLUSIONS:**

The newly proposed CRIS classification reduced the proportion of cryptogenic strokes under the TOAST system and enabled prognostic stratification by identifying a subgroup with markedly worse outcomes.

Ischemic stroke is a common and serious complication of cancer, associated with substantial morbidity and mortality.^1,2^ Stroke mechanisms in patients with cancer are heterogeneous and include conventional vascular risk factors as well as cancer-associated mechanisms, such as hypercoagulability, nonbacterial thrombotic endocarditis, tumor embolism, and treatment-related thrombosis.^1–3^ However, stroke subtypes are commonly classified using conventional etiologic systems, most often the TOAST (Trial of ORG 10172 in Acute Stroke Treatment) classification, which was not designed to account for cancer-associated mechanisms.^4^ Consequently, many strokes in patients with active cancer are categorized as cryptogenic, potentially obscuring the underlying pathophysiology and limiting prognostic stratification.^5–8^

**See related article, p 2907**

Against this background, the American Heart Association recently proposed a new etiologic framework for cancer-related ischemic stroke (CRIS), categorizing it under other determined causes.^9^ CRIS is classified as probable when accompanied by conditions suggestive of cancer-associated thrombosis, such as nonbacterial thrombotic endocarditis or disseminated intravascular coagulation (DIC), and as possible when cryptogenic stroke in patients with cancer that has spread beyond the primary site or recently progressed is accompanied by markedly elevated D-dimer levels or multifocal infarcts involving multiple vascular territories. The framework may facilitate more systematic identification of CRIS.

We previously conducted the SCAN study (Ischemic Stroke in Patients With Cancer and Neoplasia), a prospective multicenter study of acute ischemic stroke and active cancer in Japan.^8,10,11^ The SCAN database includes detailed clinical, imaging, and prognostic data, enabling reclassification according to the CRIS framework. In the present study, we investigated the characteristics and prognostic implications of the newly proposed CRIS classification using the SCAN study database.

## Methods

The data that support the findings of this study are available from the corresponding author on reasonable request. This study was conducted and reported in accordance with the Strengthening the Reporting of Observational Studies in Epidemiology guidelines.

### Standard Protocol Approvals, Registrations, and Patient Consent

This study complied with the Declaration of Helsinki for investigations involving humans and was approved by the institutional review board of The University of Osaka Hospital (approval number: 15346‐10). Written informed consent for participation in the SCAN study was obtained from all participants before enrollment.

### Study Design and Population

The SCAN study is a prospective, multicenter, observational study conducted at 9 stroke centers in Japan, between June 2016 and December 2021. Details are described in our previous publication.^8^ Briefly, patients aged ≥20 years with acute ischemic stroke within 14 days of onset and active cancer were enrolled. Active cancer was defined as cancer treated within the 6 months before admission, untreated malignancies, or metastatic disease. Among the 135 patients enrolled in the SCAN study, 132 with available plasma D-dimer data were included in this study.

### Clinical Variables

The following variables were obtained from the SCAN study database: age; sex; hypertension; dyslipidemia; diabetes; smoking status; atrial fibrillation; prior stroke; prestroke antithrombotic therapy (antiplatelet or anticoagulant); prestroke modified Rankin Scale; prestroke cancer treatment (surgery, chemotherapy, or radiotherapy); presence of distant metastasis; adenocarcinoma; National Institutes of Health Stroke Scale score; infarct pattern on brain imaging; use of r-tPA (recombinant tissue-type plasminogen activator); endovascular thrombectomy; plasma D-dimer levels; serum C-reactive protein levels; and 1-year survival after stroke onset.

Stroke subtypes classified according to the TOAST criteria were prospectively recorded in the SCAN database. For etiological classification, a standardized diagnostic work-up was applied as follows: all patients underwent blood tests, 24-hour electrocardiographic monitoring at least 7 days after admission, carotid ultrasonography, and brain imaging (computed tomography and/or magnetic resonance imaging). Transthoracic and transesophageal echocardiography was performed as necessary. In the present study, ischemic stroke subtypes were reclassified using the newly proposed CRIS criteria.^9^ Patients meeting either the probable or possible definitions were classified as CRIS. Among the 132 patients with available D-dimer data, fibrinogen levels were missing in 34 patients. Prothrombin time was not recorded in the registry. Therefore, DIC was considered present only when it had been documented as the underlying cause; otherwise, the assessment was based on the available laboratory parameters, including plasma D-dimer levels and platelet counts. In addition, the susceptibility vessel sign was not recorded in the registry. Accordingly, reclassification was performed using the remaining available diagnostic criteria.

### Statistical Analysis

Descriptive statistics were presented using the median and interquartile range (IQR) for continuous variables and as proportions and counts for categorical variables.

First, stroke subtypes initially classified according to the TOAST criteria were subsequently reclassified using the newly proposed CRIS classification. Subsequently, baseline patient characteristics were compared among conventional etiologies (small vessel occlusion, large artery atherosclerosis, cardioembolism, and others excluding CRIS), cryptogenic stroke, and CRIS. Continuous variables were compared using the Kruskal-Wallis test, and categorical variables were compared using the χ^2^ test or Fisher exact test, as appropriate. Finally, Kaplan-Meier survival curves were constructed to compare 1-year survival following stroke onset. Survival analyses were performed using both the original TOAST classification and the newly proposed CRIS classification. The 3-month survival rate was estimated, and corresponding 95% CIs were calculated.

Statistical analysis was performed using the R software (https://cran.r-project.org). The level of significance was set at *P*<0.05. All tests were 2‐tailed.

## Results

### Reclassification Using the American Heart Association-Proposed CRIS Classification

**Figure 1.**
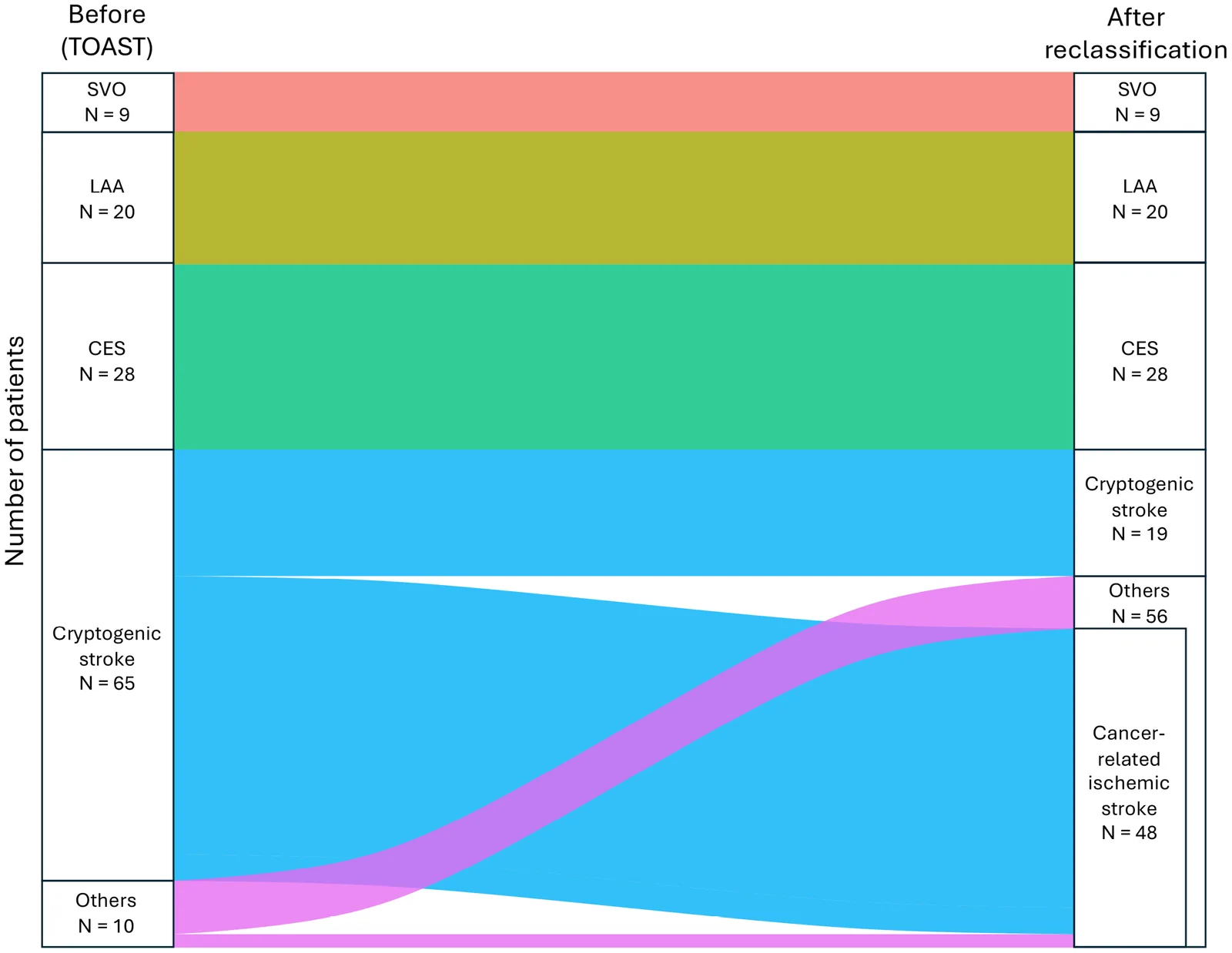
**Reclassification of ischemic stroke subtypes from TOAST (Trial of ORG 10172 in Acute Stroke Treatment) to the cancer-related ischemic stroke classification.** Sankey diagram illustrating the redistribution of ischemic stroke subtypes after applying the cancer-related ischemic stroke (CRIS) classification to patients initially classified according to the TOAST criteria. The left column represents stroke subtypes based on the TOAST classification, including small vessel occlusion (SVO), large artery atherosclerosis (LAA), cardioembolism (CES), cryptogenic stroke, and others. The right column shows the reclassified subtypes according to the CRIS classification. The width of each flow corresponds to the number of patients in each category. Notably, a substantial proportion of patients originally classified as cryptogenic stroke were reclassified as CRIS.

Among the 132 patients included in the analysis, the median age was 75 years (IQR, 69–81 years), and 50 (37.9%) were female. Figure 1 shows a Sankey diagram illustrating the reclassification of ischemic stroke subtypes from the TOAST classification to the newly proposed classification. As shown in Figure 1, 9 patients were classified as small vessel occlusion, 20 as large artery atherosclerosis, 28 as cardioembolism, 65 as cryptogenic stroke, and 10 as others under the TOAST classification. After reclassification, the number of cryptogenic stroke cases decreased to 19, with 46 patients reclassified as CRIS. In addition, 2 patients originally classified as others due to DIC were reclassified as CRIS. Among the 48 CRIS cases, 6 were classified as probable and 42 as possible CRIS. The classification of small vessel occlusion, large artery atherosclerosis, and cardioembolism remained unchanged.

### Baseline Characteristics of the Patients by Stroke Subtypes

The Table shows baseline characteristics of the patients. There were no significant differences among groups with respect to age or sex. Similarly, no significant differences were observed in the prevalence of hypertension, dyslipidemia, diabetes, or smoking status. Prestroke modified Rankin Scale scores did not differ significantly among groups. Cancer treatment tended to be more frequent in patients with conventional causes, whereas distant metastasis was more common in the CRIS group. Baseline National Institutes of Health Stroke Scale scores did not differ significantly among groups. D-dimer levels were lowest in conventional etiologies and highest in the CRIS group. A similar pattern was observed for C-reactive protein levels, which were lowest in conventional etiologies and highest in CRIS. Compared with patients classified as CRIS, those who remained classified as cryptogenic had lower D-dimer levels (3.40 [1.69–10.35] μg/mL versus 11.83 [7.99–31.36] μg/mL; *P*<0.001) and a lower frequency of infarcts involving multiple vascular territories (42.1% versus 75.0%; *P*=0.011). The detailed comparison between these 2 groups is presented in Table S1.

**Table.**
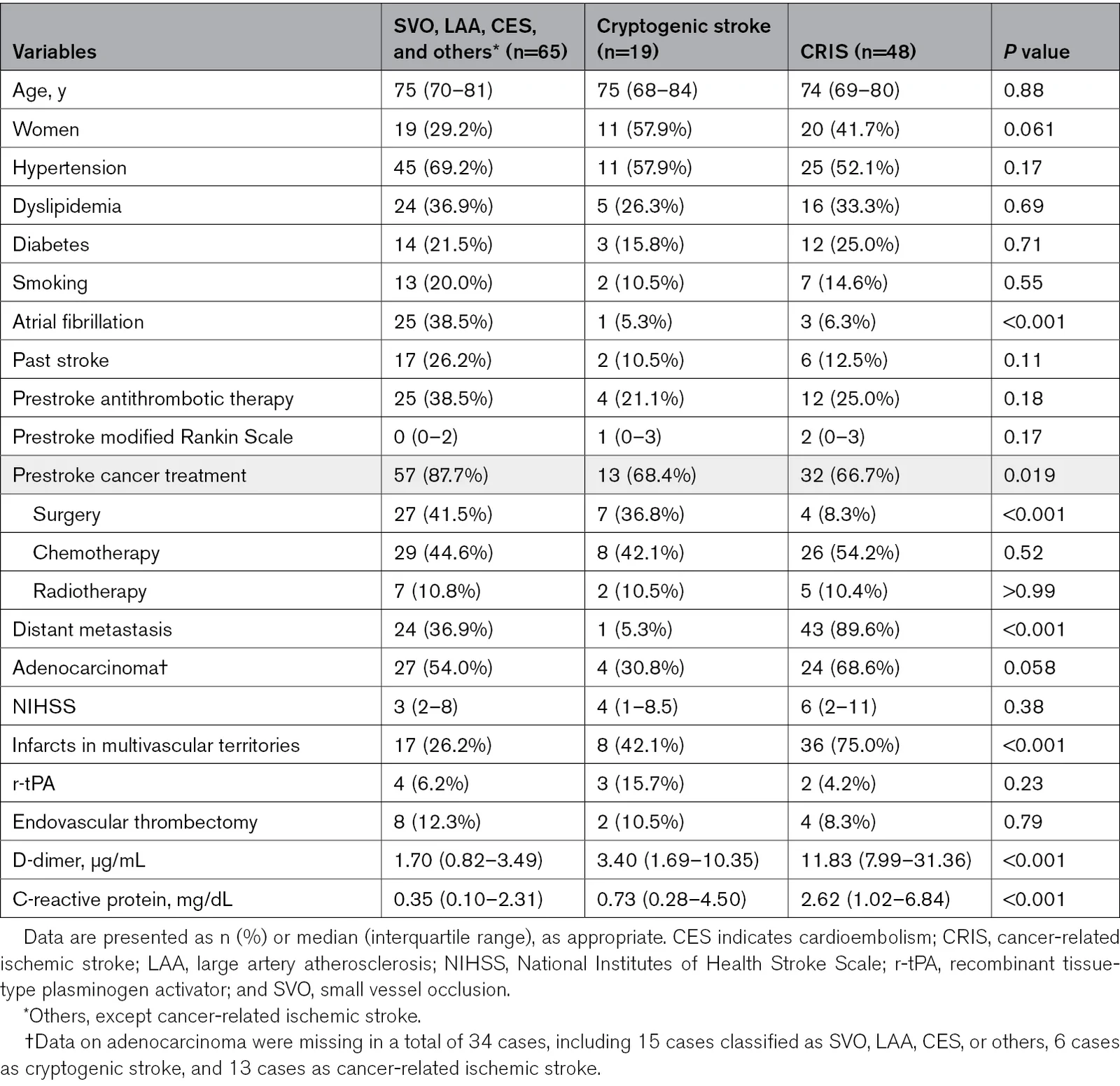
Characteristics of the Study Cohort by Stroke Subtype

### Prognostic Stratification by Reclassification Using the CRIS Framework

**Figure 2.**
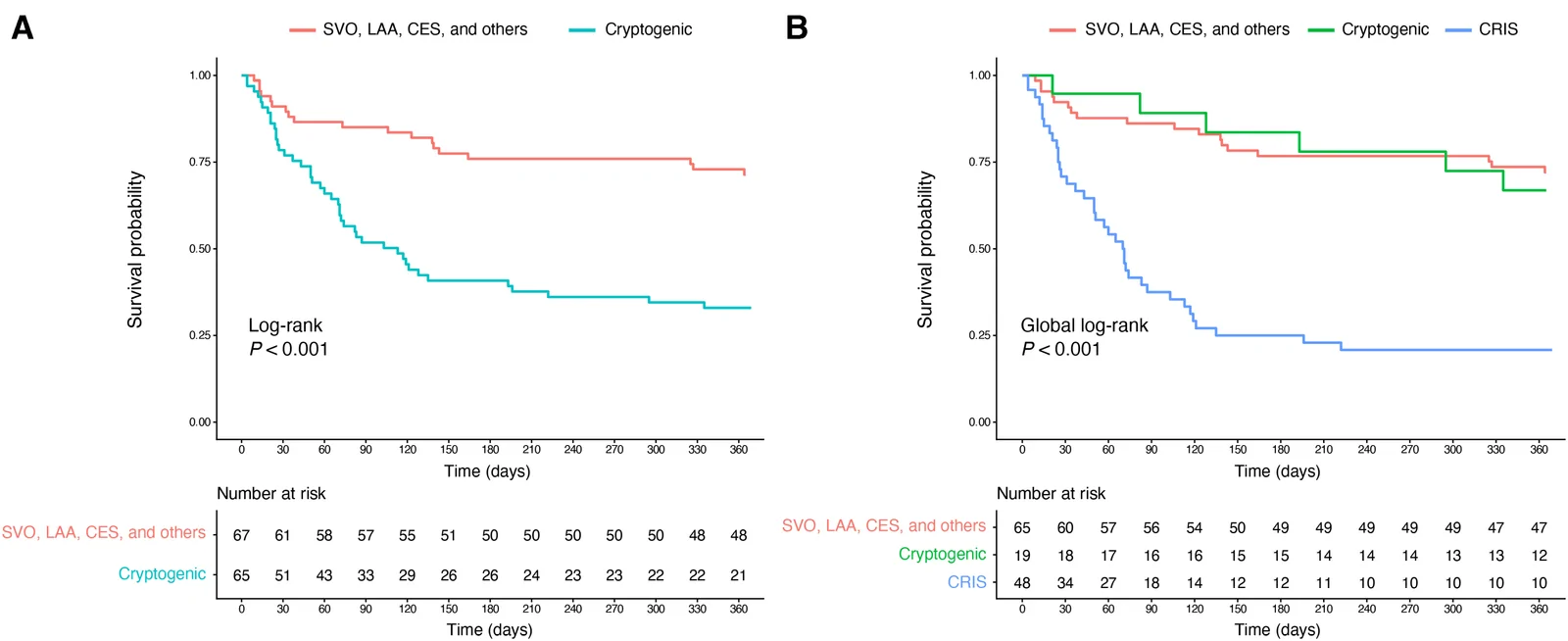
**One-year survival after stroke onset according to the TOAST (Trial of ORG 10172 in Acute Stroke Treatment) classification and the cancer-related ischemic stroke classification.** Kaplan-Meier survival curves showing 1-year survival after stroke onset. **A**, Survival according to the original TOAST classification, comparing conventional etiologies (small vessel occlusion [SVO], large artery atherosclerosis [LAA], cardioembolism [CES], and others) with cryptogenic stroke. Survival differences were assessed using the log-rank test. **B**, Survival according to the reclassified subtypes based on the cancer-related ischemic stroke (CRIS) classification, comparing conventional etiologies, cryptogenic stroke, and CRIS. Differences among the 3 groups were evaluated using the global log-rank test. In the TOAST-based analysis, patients with cryptogenic stroke had significantly poorer survival compared with those with conventional etiologies (log-rank, *P*<0.001). After reclassification, survival did not differ significantly between cryptogenic stroke and conventional etiologies; however, patients classified as CRIS demonstrated significantly worse survival (global log-rank, *P*<0.001). Numbers at risk are shown below each panel.

Figure 2 presents Kaplan-Meier survival curves based on the TOAST classification and the reclassified stroke subtypes. In analyses using the TOAST classification (Figure 2A), patients with cryptogenic stroke had significantly worse survival compared with those with known etiologies (log-rank test, *P*<0.001). In the cryptogenic stroke group, the 3-month survival rate was 51.8% (95% CI, 40.9%–65.6%). After reclassification (Figure 2B), survival did not differ between cryptogenic stroke and conventional causes during the 1-year follow-up period; however, patients classified as CRIS had significantly poorer survival (global log-rank test, *P*<0.001). In the CRIS group, the 3-month survival rate was 37.5% (95% CI, 26.0%–54.0%), whereas the corresponding 3-month survival rates were 86.2% (95% CI, 78.2%–95.0%) in patients with conventional causes (small vessel occlusion, large artery atherosclerosis, cardioembolism, and other determined cause) and 89.2% (95% CI, 76.0%–100.0%) in those with cryptogenic stroke. The median time from stroke onset to death was shortest in the CRIS group (55 days [IQR, 25–83]), compared with patients with conventional causes (90 days [IQR, 35–142]) and those with cryptogenic stroke (160 days [IQR, 94–270]). Overall, the new classification differentiated the prognosis of CRIS more distinctly than the TOAST classification.

### Antithrombotic Treatment Patterns in Patients With CRIS and Cryptogenic Stroke

Antithrombotic treatment patterns in patients with CRIS and cryptogenic stroke are presented in Figure S1. Anticoagulation was used more frequently in patients with CRIS than in those with cryptogenic stroke during the acute phase (79.1% versus 63.1%), whereas its use was similar between groups for secondary prevention (60.4% versus 57.8%). Among patients receiving antithrombotic therapy for secondary prevention, anticoagulation was used in 96.6% of patients with CRIS and 68.8% of those with cryptogenic stroke. These findings suggest that clinicians preferentially selected anticoagulation in cases clinically suspected to be cancer-related, and that anticoagulation was used in more than half of patients with cryptogenic stroke.

## Discussion

In this study, reclassification using the CRIS framework reduced cryptogenic stroke from 49.2% to 14.4% and identified 36.3% of patients as having CRIS. Patients remaining cryptogenic after reclassification had survival comparable to those with conventional stroke mechanisms, whereas patients with probable or possible CRIS had significantly worse outcomes. These findings suggest that the CRIS framework captures clinically meaningful prognostic differences among patients with acute ischemic stroke and active cancer.

The clinical significance of our findings lies in the ability of the CRIS framework to distinguish clinically meaningful subgroups among patients previously classified as cryptogenic by the TOAST classification.^4–8^ Because TOAST lacks criteria for cancer-associated mechanisms, many patients with cancer-associated stroke are categorized as cryptogenic. By applying the CRIS framework, we identified a high-risk subgroup of patients meeting probable or possible CRIS criteria with markedly worse outcomes, whereas patients remaining cryptogenic showed survival comparable to conventional stroke mechanisms. These findings suggest that the CRIS framework helps formalize clinical intuition into a systematic classification for patients with cancer.

Some conceptual considerations may be relevant to the classification of CRIS. First, DIC is included as a mechanism underlying CRIS, and the scientific statement proposes applying the International Society on Thrombosis and Haemostasis criteria for its diagnosis.^12^ However, in solid malignancies, coagulation abnormalities are often characterized by a chronic and relatively low-grade activation rather than an acute consumptive process.^13^ Although malignancy is included among the conditions associated with overt DIC in the International Society on Thrombosis and Haemostasis criteria, these criteria also apply to acute systemic conditions such as sepsis and trauma.^12^ As such, whether they adequately reflect the chronic and often low-grade coagulation abnormalities observed in patients with cancer remains uncertain. Second, because the CRIS framework partly relies on tumor progression beyond the primary site or recent progression, some patients with early-stage malignancy and cancer-associated thrombosis may not meet the criteria despite likely cancer-associated mechanisms. It has been reported that some patients with localized cancer exhibit only modest elevations in D-dimer levels.^14^ These considerations suggest that, although the CRIS framework provides a structured approach to identifying CRIS, some ambiguity remains. Future studies are warranted to refine the criteria and improve clinical applicability.

This study has several limitations. First, although the SCAN study was prospective and multicenter, this analysis was retrospective, and not all CRIS criteria were available. Variables required for DIC diagnosis, locally progressive cancer, or susceptibility vessel sign were unavailable, which may have resulted in misclassification. Second, the sample size was relatively small, particularly after subgroup stratification, limiting statistical power. Finally, because patients were enrolled from Japanese stroke centers, generalizability may be limited. Future prospective studies are needed to validate this framework.

In conclusion, because cancer-associated coagulopathy is often chronic rather than acute, the applicability of the International Society on Thrombosis and Haemostasis criteria remains uncertain, and some patients with early-stage cancer may not meet the current CRIS criteria despite likely cancer-associated mechanisms. Nevertheless, the CRIS framework stratified cryptogenic stroke into distinct prognostic groups. Patients classified as probable or possible CRIS had worse outcomes, whereas those remaining cryptogenic showed survival comparable to conventional causes. These findings support the clinical relevance of the CRIS framework.

## Article Information

### Acknowledgments

The authors would like to thank all investigators for their efforts in conducting the SCAN study (Ischemic Stroke in Patients With Cancer and Neoplasia).

### Disclosures

None.

### Supplemental Material

Table S1

Figure S1

STROBE Checklist

## Supplementary Material


